# Boerhaave Syndrome Caused by Pancreatitis-Induced Gastric Outlet Obstruction: A Case Report

**DOI:** 10.7759/cureus.115701

**Published:** 2026-09-03

**Authors:** Victor Viegas, Hugo Pais Moreira, Inês Sousa, Daniel Martins, Andreia Amado, Mariana Santos, Amélia Tavares, Miguel Mendes, Fernando Viveiros, Bela Pereira

**Affiliations:** 1 General Surgery, Unidade Local de Saúde Gaia e Espinho, Vila Nova de Gaia, PRT

**Keywords:** acute pancreatitis, boerhaave syndrome, emergency, esophageal perforation, surgery

## Abstract

Boerhaave syndrome is a spontaneous transmural perforation of the esophagus, typically triggered by forceful vomiting and carrying a high mortality rate when diagnosis is delayed. We present a 56-year-old man with chronic alcohol use disorder who developed esophageal perforation through an unusual causal chain. He presented with intractable vomiting, hematemesis, and cervical subcutaneous emphysema. Computed tomography showed posterior pneumomediastinum and marked gastric and duodenal distention caused by an inflammatory cephalopancreatic mass. Elevated pancreatic enzymes confirmed acute pancreatitis. The sequence appeared to be alcoholic pancreatitis compressing the duodenum, causing gastric outlet obstruction, protracted emesis, and esophageal rupture. On the day of admission, laparoscopic primary two-layer esophagorrhaphy and gastrojejunostomy were performed to address both the perforation and the duodenal obstruction. The postoperative course was complicated by recurrent pancreatitis and bilateral pleural effusions, but serial imaging confirmed no leak at the esophageal repair or the anastomosis. The patient was discharged on postoperative day 25. Follow-up magnetic resonance cholangiopancreatography excluded pancreatic malignancy and showed findings consistent with groove pancreatitis and chronic pancreatitis. This case illustrates an unusual causal chain in which acute pancreatitis led to esophageal perforation via gastric outlet obstruction and reinforces the benefit of same-day surgical repair when both the perforation and its cause can be addressed in a single procedure.

## Introduction

Boerhaave syndrome is defined as a spontaneous, transmural perforation of the esophagus, most commonly triggered by forceful emesis [1]. With an estimated annual incidence of approximately 3.1 per 1,000,000 population, it remains one of the rarest and most lethal gastrointestinal emergencies [2]. Even with intervention, pooled mortality remains approximately 12% in contemporary series, driven largely by diagnostic delay and mediastinal contamination [1,3]. The perforation typically occurs in the left distal esophagus, where the muscular wall is thinnest [2].

The Mackler triad (vomiting, lower thoracic pain, and cervical subcutaneous emphysema) is present in only 20% to 50% of cases, which frequently delays diagnosis [2]. In most reported cases, protracted vomiting is related to excessive alcohol or food intake [4]. A less frequently reported cause of oral intolerance is gastric outlet obstruction from acute pancreatitis [5].

We report a case of Boerhaave syndrome in a 56-year-old man with chronic alcohol use disorder, in whom the esophageal perforation followed intractable vomiting attributed to duodenal obstruction from pancreatic inflammation. We propose, as the most plausible mechanism rather than a definitively proven one, an indirect causal chain in which alcohol-related pancreatitis produced gastric outlet obstruction, protracted emesis, and ultimately transmural esophageal rupture; alcohol may contribute both to pancreatic inflammation and to impaired tissue healing.

## Case presentation

A 56-year-old man with a history of chronic alcohol use disorder and hepatic steatosis (last hepatology follow-up approximately eight years earlier) presented to the emergency department. He reported recurrent vomiting since the previous day, with progressive worsening and hematemesis. He also described intense abdominal pain of sudden onset. His regular medication consisted of alprazolam 0.5 mg daily. He denied respiratory symptoms and chest pain. The full clinical course is summarized in Table 1.

**Table 1:** Clinical timeline relative to admission (POD, with the operation on the day of admission). POD, postoperative day; NPO, nil per os; PPI, proton pump inhibitor; CT, computed tomography; MRCP, magnetic resonance cholangiopancreatography; CEA, carcinoembryonic antigen; CA 19-9, carbohydrate antigen 19-9; ref, reference interval.

Timepoint	Clinical events and management	Investigations and key findings
Day before admission	Onset of recurrent, progressively worsening vomiting	-
Day 1 (admission)	Presentation: protracted vomiting, hematemesis, sudden severe abdominal pain, cervical and upper-thoracic subcutaneous emphysema. Started NPO, IV esomeprazole 80 mg, piperacillin-tazobactam, analgesia	Leukocytosis 16.08 x10³/µL; amylase 180 U/L (ref 13-53), lipase 232 U/L (ref 3-60); CRP 0.39 mg/dL. CT #1: posterior pneumomediastinum; gastric and D1-D2 distension; cephalopancreatic mass; bilateral pleural effusion; main pancreatic duct dilation
Day 1 (operation, POD 0)	Laparoscopic two-layer primary esophagorrhaphy and posterior mediastinal drain; gastrojejunostomy for duodenal obstruction	Intraoperative: full-thickness distal esophageal laceration (3 cm mucosal, 5 cm muscular); obstructive duodenal lesion from cephalopancreatic pathology confirmed
POD 0-3	NPO, nasogastric drainage (~750 mL/day), total parenteral nutrition, double-dose IV PPI, antiemetics, prokinetics, piperacillin-tazobactam and fluconazole	-
POD 3	Nasogastric output tapered, tube removed; liquid oral diet introduced and tolerated	Negative blood cultures, urine cultures, and respiratory virus panel
POD 7	Oral diet advanced	CT #2 (oral contrast): no leak at esophageal repair or anastomosis; marked improvement of pneumomediastinum; uncinate lesion ~47 x 32 mm with calcifications, double duct sign, 12-mm inter-portocaval node; incidental left-upper-lobe nodule. Tumor markers normal (CEA 1.7 ng/mL, CA 19-9 9.5 U/mL)
POD 13	Fever 38 °C, recurrent vomiting, epigastric pain; returned to NPO and parenteral nutrition	Biochemical relapse: amylase 854 U/L, lipase 1448 U/L. CT #3: repair and anastomosis intact; pattern favors groove pancreatitis over neoplasm
POD 18	Empirical antibiotic escalation to imipenem for persistent systemic inflammation despite piperacillin-tazobactam	-
POD 20	Tube thoracostomy for right pleural loculation	CT #4: right pleural superinfection with loculated basal effusion (66 x 54 mm) and peripancreatic collection (42 x 24 mm); no esophageal or anastomotic leak; no pancreatic mass
POD 20-25	Progressive recovery; fevers resolved, inflammatory markers and pancreatic enzymes downtrended; chest drain removed; diet reintroduced (liquids then low-fat solids)	-
POD 25	Discharge with transhiatal drain in situ	-
~1 week post-discharge	Transhiatal drain removed at outpatient visit	-
~3 months after admission	Follow-up	MRCP: chronic pancreatitis with residual groove-pancreatitis collection; no pancreatic malignancy

On examination, the patient was agitated and diaphoretic. His temperature was 36 °C, blood pressure was 149/98 mmHg, and heart rate was 110 beats per minute. The Glasgow Coma Scale score was 15. Subcutaneous emphysema was noted on palpation of the cervical and upper thoracic regions. The abdomen was tense and diffusely tender, with guarding on palpation and pain rated 7 out of 10.

Initial laboratory workup showed leukocytosis (16.08 x 10³/µL, 61.7% neutrophils), elevated pancreatic enzymes (amylase 180 U/L, reference 13-53 U/L; lipase 232 U/L, reference 3-60 U/L), elevated gamma-glutamyltransferase (225 U/L) and alkaline phosphatase (173 U/L), and C-reactive protein of 0.39 mg/dL. Cardiac biomarkers were within normal limits. An arterial blood gas analysis on room air showed mild hypoxemia (PaO₂ 65.5 mmHg, SaO₂ 94.5%) with lactate of 1.6 mmol/L. An electrocardiogram showed no acute ischemic changes.

A first contrast-enhanced thoracoabdominal computed tomography (CT) scan revealed pneumomediastinum confined to the posterior mediastinum (Figure 2B), suggestive of esophageal perforation, although no definite perforation site was identified. There was no pneumoperitoneum. Other findings included a thin bilateral pleural effusion, interpreted as reactive and not requiring drainage, and diffuse dilation of the main pancreatic duct suggestive of underlying chronic pancreatitis. The general surgery team reviewed the imaging and additionally noted marked distention of the stomach and the first and second portions of the duodenum (Figure 1A and Figure 2A), attributed to an indeterminate mass in the cephalopancreatic region (Figure 1B and Figure 2B). 

**Figure 1:**
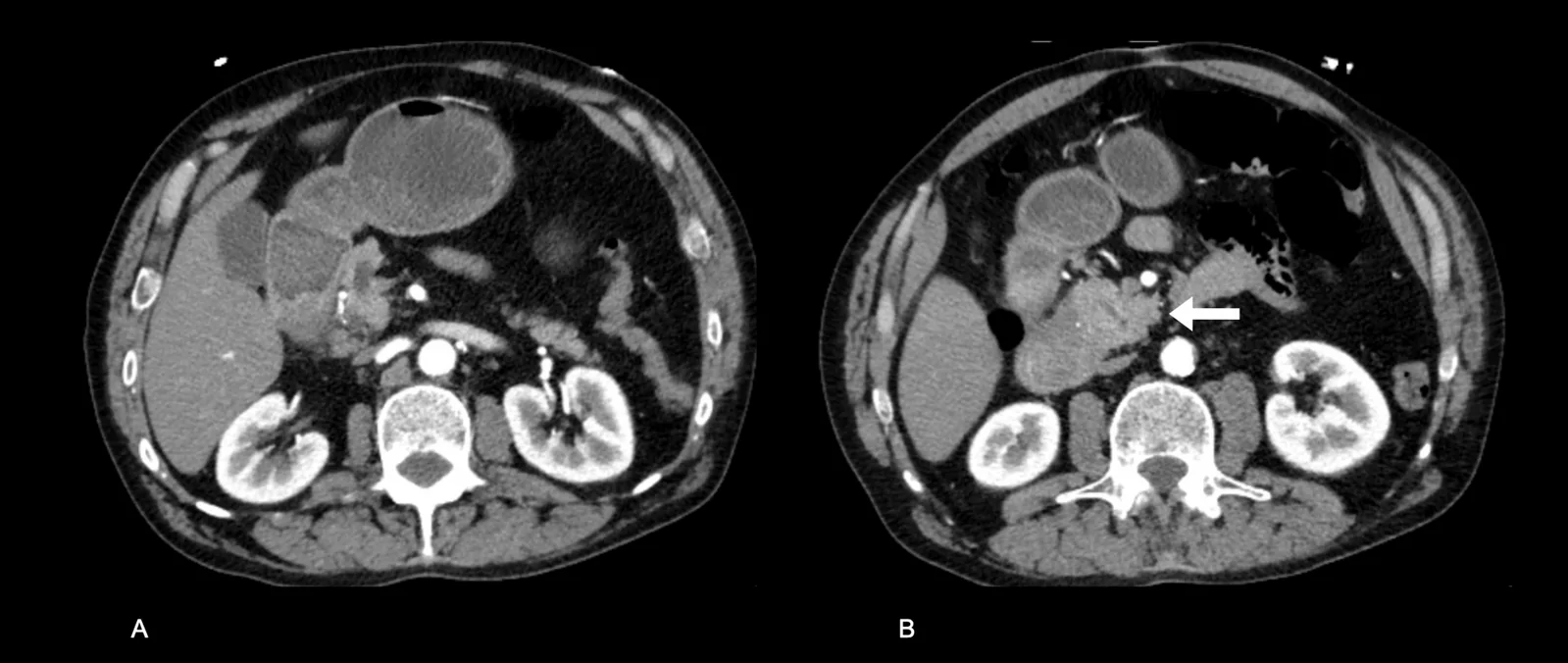
Contrast-enhanced axial CT of the upper abdomen at admission (portal venous phase). (A) Axial image at the level of the pancreatic head showing marked distention of the stomach and the first and second portions of the duodenum. (B) Slightly caudal axial image showing a heterogeneous cephalopancreatic lesion (white arrow) with loss of the fat plane between the lesion and the adjacent duodenum.

**Figure 2:**
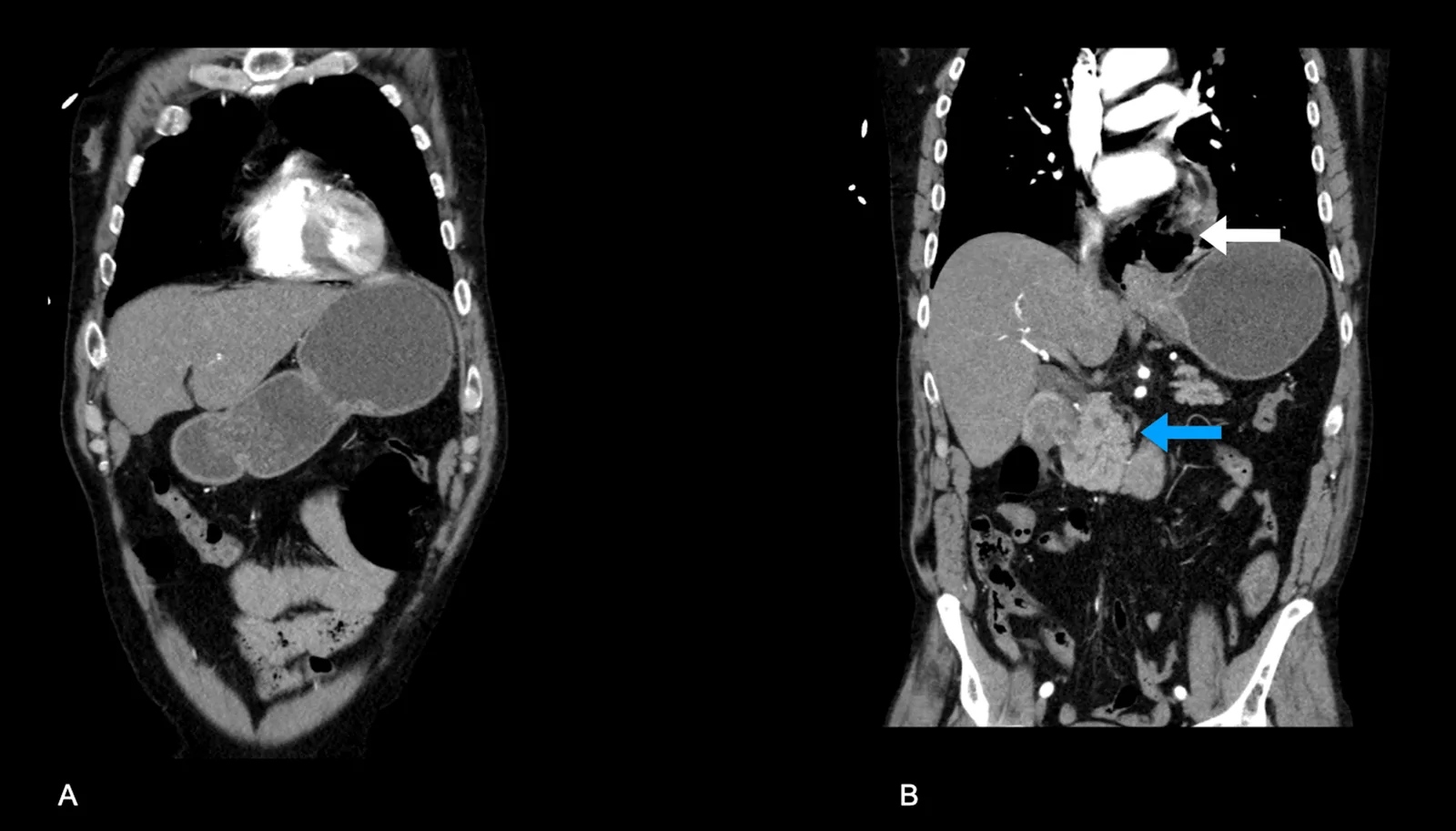
Contrast-enhanced coronal CT of the chest and abdomen at admission (portal venous phase). (A) Coronal image showing a massively distended, fluid-filled stomach with dilated duodenal loops. (B) Posterior coronal image showing posterior pneumomediastinum (white arrow) and the cephalopancreatic mass narrowing the duodenum (blue arrow); in grayscale versions, the white arrow is the superior (thoracic) marker and the blue arrow is the inferior (pancreatic) marker.

The clinical and radiological findings were considered consistent with Boerhaave syndrome. However, the cephalopancreatic mass causing duodenal obstruction raised concern for a pancreatic malignancy that could not be excluded at that time. The patient was initially managed with nil per os, an intravenous proton pump inhibitor (esomeprazole 80 mg), broad-spectrum antibiotics (piperacillin-tazobactam), and analgesia. At presentation, the patient's Pittsburgh perforation severity score (PSS) was 5, comprising tachycardia (heart rate >100/min, 1 point), leukocytosis (>10,000/µL, 1 point), pleural effusion (1 point), and a noncontained perforation (2 points); the remaining components (age >75 years, fever, respiratory compromise, time to diagnosis >24 h, hypotension, and malignancy) were absent. On the PSS (range 0-18), a score of 5 corresponds to the intermediate-risk stratum (3-5); scores of 2 or less are generally associated with successful nonoperative or endoscopic management, whereas scores above 5 carry substantially higher morbidity and mortality and more often require operative treatment [6]. Management, however, depends not on the score alone but also on perforation site, containment, contamination, physiological stability, the underlying pathology, time to diagnosis, and local expertise. In this patient, an intermediate score alone might have permitted consideration of a nonoperative or endoscopic approach, but this was not viable: the gastric outlet obstruction from pancreatic inflammation remained unaddressed, and without relieving it, the mechanism driving the perforation would persist. Operative intervention was therefore pursued to manage both pathologies in the same setting.

The patient was taken to the operating room on the day of admission. A diagnostic laparoscopy was performed using five ports (three 5-mm, one 10-mm, and one 12-mm trocar), with pneumoperitoneum established at Palmer’s point. Inspection of the esophageal hiatus confirmed the integrity of the diaphragmatic crura. After opening the phrenoesophageal ligament and the pars flaccida of the hepatogastric ligament, a small amount of saliva and clots was found draining from the posterior mediastinum. The distal esophagus was mobilized, and a full-thickness esophageal laceration was identified on the anterior wall, just above the gastroesophageal junction, with a mucosal tear of approximately 3 cm and a muscular tear of approximately 5 cm (Figure 3A). The mediastinal contamination was limited. Primary repair of the esophageal laceration was performed laparoscopically in two layers using continuous Stratafix 3/0 sutures, one for the mucosal layer and another for the muscularis propria (Figure 3B). The posterior mediastinum was irrigated with normal saline. A 19-French Blake drain was placed in the posterior mediastinum in a retroesophageal position, coursing through a subhepatic trajectory and exteriorized through the right flank. No intraoperative leak test was performed, and no tissue flap or buttress was used, given the limited mediastinal contamination and viable tissue edges.

**Figure 3:**
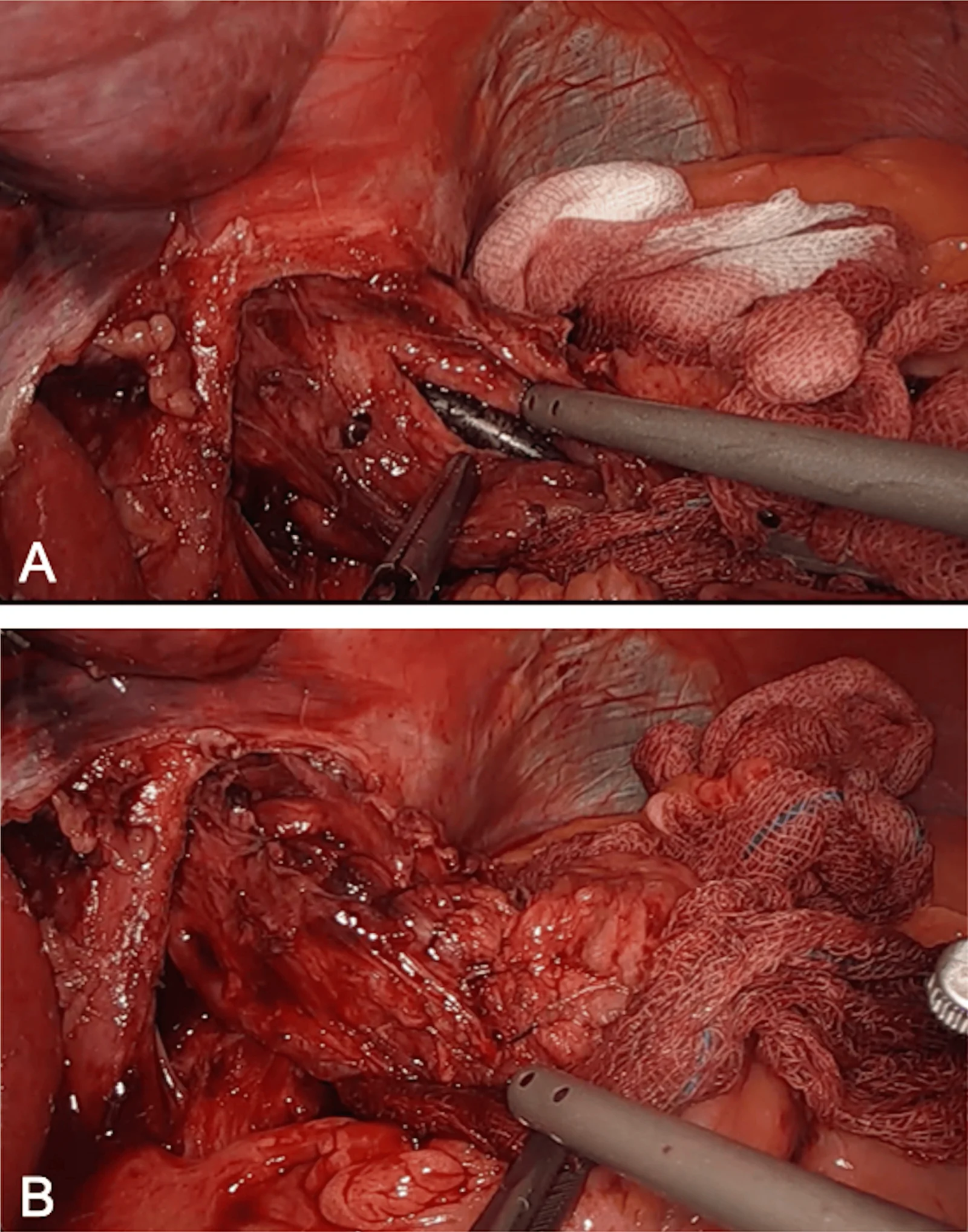
Intraoperative laparoscopic views of the distal esophagus. (A) Full-thickness laceration of the anterior esophageal wall just above the gastroesophageal junction; the disrupted mucosal and muscular edges lie at the centre of the field, with a nasogastric tube visible within the lumen. (B) Completed two-layer primary repair; the continuous Stratafix 3/0 suture line runs along the anterior esophageal wall, with the gastroesophageal junction inferiorly.

Intraoperatively, an obstructive lesion at the level of the duodenum, attributed to cephalopancreatic pathology, was also identified, confirming a mechanical gastric outlet obstruction. A laparoscopic gastrojejunostomy was constructed as a side-to-side anastomosis to the posterior wall of the stomach using a 60-mm linear stapler with 3-mm staples. The enterotomy was closed with a continuous Stratafix 2/0 suture.

The postoperative course was managed in the surgical ward. The patient was kept nil per os with nasogastric tube drainage and started on total parenteral nutrition, a double-dose intravenous proton pump inhibitor, antiemetics, prokinetics, and empirical broad-spectrum antibiotic coverage with piperacillin-tazobactam 4.5 g (17 days) and fluconazole 200 mg (25 days), the latter added because upper gastrointestinal perforations carry a high risk of Candida contamination. The mediastinal drain initially produced serosanguineous fluid, and the nasogastric tube drained roughly 750 mL per day.

The first days were encouraging. By postoperative day 3, nasogastric output had tapered, the tube was clamped and then removed, and a liquid oral diet was introduced and tolerated without nausea or vomiting. Blood cultures, urine cultures, and respiratory virus panels were negative. With clinical and biochemical recovery underway, a second contrast-enhanced thoracoabdominal CT with oral contrast was performed on postoperative day 7 to confirm the integrity of the esophageal repair and the gastrojejunostomy before advancing the diet further. The study showed no contrast extravasation at either site and marked improvement of the pneumomediastinum, with only a 9-mm residual gas bubble adjacent to the gastroesophageal junction. It also revealed a previously unappreciated finding: a heterogeneous, ill-defined solid lesion measuring approximately 47 x 32 mm at the uncinate process of the pancreas, containing three millimetric calcifications, with loss of fat planes against the duodenal wall and a suspicious 12-mm inter-portocaval lymph node. Dilation of the intrahepatic biliary tree and the common bile duct (9 mm), together with dilation of the main pancreatic duct up to 7 mm, produced a classic “double duct sign.” An incidental 24 x 24 mm nodular consolidation was also noted in the left upper lobe and referred for outpatient follow-up; it was subsequently investigated by pulmonology and diagnosed as noncavitary pulmonary tuberculosis, for which antituberculous therapy was initiated. At this point, the differential lay between a pancreatic head neoplasm and focal pancreatitis with a pseudotumoral appearance. Tumor markers were reassuring (CEA 1.7 ng/mL, CA 19-9 9.5 U/mL).

On postoperative day 13, the patient developed a fever of 38 °C, oral intolerance with recurrent vomiting, and epigastric pain. A third CT was obtained the same day to exclude an intra-abdominal collection, an anastomotic complication, or progression of the suspected pancreatic lesion. The esophageal repair and the gastrojejunostomy remained intact. The pancreatic head was now globular and ill-defined, but no discrete mass was identifiable on this protocol. Critically, new findings emerged: dense peripancreatic fat stranding extending to the hepatic hilum, parietal thickening with microcystic change in the first and second duodenal portions, and inflammatory wall changes of the common bile duct. The radiologic pattern now favored groove pancreatitis over a neoplastic process. This shift was supported by a sharp biochemical relapse: amylase rose to 854 U/L and lipase to 1448 U/L, consistent with recurrent acute pancreatitis. The oral diet was withheld, and the patient was returned to nil per os with parenteral nutrition.

Over the following days, the pancreatic flare, rather than the esophageal repair, became the dominant clinical problem, with intermittent low-grade fevers and persistently elevated inflammatory markers despite piperacillin-tazobactam. Antibiotic therapy was therefore empirically escalated to imipenem 500 mg on postoperative day 18 (continued for 8 days), chosen for its broad Gram-negative and anaerobic spectrum, adequate penetration into pancreatic tissue and pleural fluid, and activity against the resistant Enterobacterales typically implicated in nosocomial intra-abdominal and pleural infection. A fourth CT on postoperative day 20 then searched for an undrained septic focus, either an intra-abdominal collection or a complicated pleural process, and reassessed the evolution of the pancreatic disease. It showed pleural thickening and hyperenhancement compatible with pleural superinfection, a new 66 x 54 mm loculated right basal effusion, and a new 42 x 24 mm peripancreatic fluid collection, but no esophageal or anastomotic leak and no defined pancreatic mass. The right pleural loculation prompted tube thoracostomy. Pleural-fluid cultures were negative, and drainage was limited (approximately 20-25 mL); infection was therefore suspected radiologically but not microbiologically confirmed.

From this point, the patient improved steadily. Fevers resolved, inflammatory markers and pancreatic enzymes downtrended, and the chest drain was removed once output diminished. The diet was reintroduced cautiously, first as liquids and then as a low-fat solid diet, and he was discharged on postoperative day 25 with the transhiatal drain in place; this was removed at the outpatient visit one week later.

A follow-up magnetic resonance cholangiopancreatography approximately three months after admission clarified the underlying disease (Figure 4). It showed parenchymal atrophy of the pancreatic body and tail with irregular dilation of the main pancreatic duct (up to 6 mm) and side-branch dilation, consistent with chronic pancreatitis. An 18-mm multiloculated lesion with an enhancing wall and restricted diffusion sat in the pancreatoduodenal groove in contact with the second duodenal portion, most consistent with a residual collection from groove pancreatitis. The bile ducts were mildly dilated (CBD 8 mm) without choledocholithiasis. No pancreatic malignancy was identified. Tissue diagnosis had not been pursued at the emergent index operation, where biopsy of an acutely inflamed pancreatic head would have carried a high risk of fistula and hemorrhage with an unreliable frozen section; malignancy was instead evaluated noninvasively, with normal tumor markers and interval regression of the cephalopancreatic lesion, which was no longer identifiable on later imaging. This course argues strongly against neoplasia and supports an inflammatory pseudotumor of groove pancreatitis as the most likely basis for the admission and early postoperative findings, although malignancy could not be excluded histologically.

**Figure 4:**
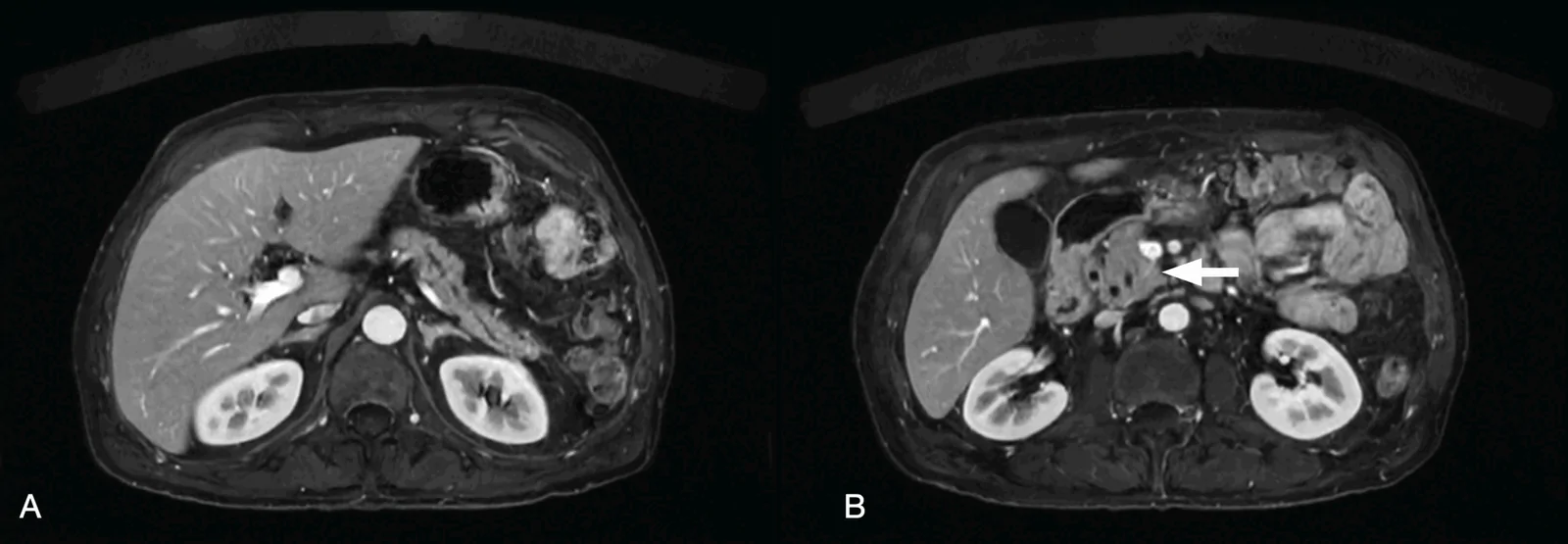
Contrast-enhanced axial magnetic resonance images from the follow-up MRCP examination (approximately three months after admission). (A) Axial image through the pancreatic body and tail showing parenchymal atrophy and ductal irregularity consistent with chronic pancreatitis. (B) Axial image through the pancreatic head; the white arrow indicates the pancreaticobiliary junction and adjacent groove region, which shows no discrete solid mass. MRCP: magnetic resonance cholangiopancreatography.

## Discussion

Boerhaave syndrome accounts for approximately 15% of all esophageal perforations and has a reported mortality rate of 30% to 90%, depending on the source [2]. The mechanism involves a sudden rise in intraesophageal pressure exceeding 150 mmHg during forceful vomiting, resulting in a full-thickness tear at the weakest point of the esophageal wall, typically the left distal third [2]. Kuwano et al. proposed that esophageal perforation results from retrograde gastrointestinal motor contraction causing gastric fornix prolapse into the esophagus, with subsequent longitudinal stretching and destruction of the muscularis mucosa [4]. In the present case, this mechanism was amplified by gastric outlet obstruction: the pancreatic inflammatory mass compressed the duodenum, preventing gastric emptying and generating forceful, repetitive emesis.

Acute pancreatitis causing gastric outlet obstruction has been documented, though primarily as a late complication. Gastric outlet obstruction complicating acute pancreatitis has been reported in approximately 6% of cases and has been managed with EUS-guided gastrojejunostomy, as described by Fuentes-Valenzuela et al. [5]. Sugimoto et al. found that 15% of patients with pancreatic duct disruption developed gastric outlet obstruction at a median of 92 days after onset [7]. In our patient, the obstruction occurred during the acute phase, with imaging revealing marked distension of the stomach and proximal duodenum alongside an inflammatory cephalopancreatic mass. The causal chain of alcoholic pancreatitis causing retroperitoneal and duodenal inflammation, leading to obstruction, protracted vomiting, and esophageal perforation has not been previously described as a unified sequence.

The diagnostic workup relied on CT, which has a reported sensitivity exceeding 90% for esophageal perforation and is now considered the preferred initial imaging modality [8,9]. Wei et al. demonstrated that CT has a negative predictive value of 100% [8]. In the present case, CT simultaneously identified the esophageal perforation (posterior pneumomediastinum) and the underlying cause (gastroduodenal distension and the cephalopancreatic inflammatory mass), enabling a single imaging study to guide the entire surgical plan.

Regarding management, a meta-analysis by Biancari et al. (75 studies, 2,971 patients) showed that primary repair carries a pooled mortality of 9.5%, compared with 7.3% for esophageal stenting and 20% for T-tube drainage [3]. Pan et al., in a more recent meta-analysis (46 studies, 944 patients), reported 6% mortality after primary closure and 8% after stent placement, with treatment initiated within 24 hours reducing mortality to 3% versus 12% when delayed [10]. The Pittsburgh perforation severity score was developed to stratify patients into risk groups and guide treatment decisions [6]. In our patient, urgent surgical intervention with primary two-layer esophagorrhaphy was chosen given the extent of contamination and the concurrent need for gastrojejunostomy to bypass the obstructed duodenum. This combined approach addressed both the perforation and its underlying cause in a single procedure.

Chronic alcohol use disorder in this patient served a dual pathogenic role: it was the etiological agent for pancreatitis [11,12] and is also a recognized risk factor for postoperative morbidity in esophageal surgery, including increased rates of pneumonia, sepsis, and mortality [13]. Despite these risk factors, the patient recovered well, likely because of early diagnosis and same-day surgical intervention. This is consistent with data showing that treatment within 24 hours significantly reduces mortality [1,3,10].

This case has limitations inherent to all single case reports: causality between pancreatitis-induced obstruction and esophageal perforation cannot be definitively established, the data are retrospective, and the findings may not be generalizable. Nevertheless, the temporal sequence and anatomical findings strongly support the proposed mechanistic chain. Malignancy was not excluded histologically; this conclusion rests on the interval regression of the pancreatic lesion, normal tumor markers, and the characteristic imaging evolution rather than on tissue confirmation.

## Conclusions

This case illustrates that Boerhaave syndrome can arise through an uncommon pathway in which pancreatitis-related inflammatory obstruction of the duodenum leads to protracted vomiting and esophageal perforation; in this patient, this sequence was the most likely explanation rather than a definitively established one. In patients with pancreatitis and intractable emesis, clinicians should consider concurrent esophageal injury, particularly when subcutaneous emphysema or pneumomediastinum is present on imaging. In this patient, early recognition and same-day operative repair with simultaneous relief of the obstruction were followed by a favorable, though protracted and complicated, recovery. As a single case, this report cannot establish the general effectiveness of combined repair and gastrojejunostomy, and these observations should be interpreted as case-specific.
